# Genito-Urinary Surgery

**Published:** 1905-12-16

**Authors:** 


### Progress in Medicine and Surgery.

### GENITO-URINARY SURGERY.

**Tuberculosis of the Kidney.**—Howard A. Kelly[1] has recently discussed this disease and its treatment. There are three possible avenues by which the disease may reach the kidneys: by the blood, by ascending the ureter, or by direct extension from a continguous organ. Baumgarten, by the experimental inoculation of the urethra in rabbits, was able to produce tuberculous ulceration of the bladder and the prostatic urethra, but could not produce tuberculosis of the kidneys nor of the vasa deferentia or testicles. He concluded that tubercle bacilli did not advance against the current of secretion. But, though this dictum is generally true, there are good clinical reasons for believing that in man the disease may extend from the testicle to the bladder and then to the kidney after producing stricture of a ureteral orifice and slight hydro-ureter. Kelly writes, "without any doubt the infection of the kidney is almost always hematogenous." The blood often becomes infected from a thoracic focus. Kelynack found the lungs affected in 70 per cent. of a series of cases examined post-mortem. A cheesy bronchial gland may be the primary focus. Disposition of the kidney for tuberculosis may be the result of trauma, of calculous disease or of hydronephrosis. The disease once having started in the kidney is, with perhaps the rarest exceptions, progressive. But a seeming cure may be obtained with sequestration of the kidney and obliteration of the lumen of the ureter. In most cases the disease at its onset is local and unilateral, and so may remain for months or even years. The disease having started in one kidney attacks in order the ureter of that side, the bladder, and usually, as a late secondary affection, the other kidney. The diagnostician has to determine not only the presence of the disease, but its extent and whether the other kidney is able to do the necessary excretory work if the diseased one is removed. It is important to discover if one or both ureters are thickened as felt by vaginal palpation. Pressure upon the enlarged cord-like ureter in the antero-lateral vaginal fornix often induces an intense desire to urinate. On the other side, usually, the normal ureter can be felt "slipping like a soft wet string between the fingers." The injection of tuberculin is valuable for diagnosis if there is marked pain produced in the kidney as well as fever. Any persistent acid pyuria, even of mild grade, which does not yield an efflorescence of organisms in culture media, must be at once suspected to be of tubercular origin. Also slight persistent cystitis, rebellious to treatment, should be suspected. Kelly prefers the direct aëroscopic examination of the bladder to other methods of cystoscopy. The ureteral orifices can be cleansed before catheterisation and hard or flexible catheters used in this method. The signs of unilateral disease are injection or ulceration of one ureteral orifice or retraction of the orifice or its appearance as a deep hole. In treatment the author recommends complete nephrectomy unless the disease is evidently limited to one pole of the kidney as demonstrated by splitting the organ from end to end. Nephrotomy as a preliminary to radical operation is a valuable procedure. It can be done under gas in three to five minutes. In old suppurative cases intracapsular enucleation is recommended as being simple and satisfactory, and far less hazardous than an ordinary nephrectomy. The author advises caution in adding ureteral extirpation to that of the kidney. The ureter may be thickened from ureteritis which is not tubercular. If disease remains rebellious in the bladder portions of that viscus may be excised.

**General Cystic Disease of the Kidneys** is a subject of much interest and importance from the facts that the diagnosis is often obscure and the principles of treatment are not adequately recognised. Edgar L. Collis and John T. Hewetson[2] have recently considered the subject in connection with a report of two patients affected with the disease who were brother and sister. The first case was a woman of thirty-eight. Eight years before her illness she had passed three urinary calculi. For about five years she had been subject to severe migrainous headaches. For a month she had frequency of micturition, and for a fortnight paroxysmal attacks of renal colic. A large irregular swelling in the left renal region proved at an operation to be a large cystic kidney and was removed. At the time of operation the right kidney was about twice the normal size. Five days after the nephrectomy (left) the right kidney had much increased in size and felt irregular, and later this kidney was about four times the natural size, and presented irregularities due, no doubt, to general cystic disease. The enlarged kidney continues to perform its functions fairly well and the patient is able to do her housekeeping work three and a half years after the operation, though she has many of the symptoms of chronic Bright's disease. In the second case, also treated by nephrectomy, there had been paroxysmal pain in the affected side of the abdomen, and for a few months before operation there was constant pain and jaundice, and a large tumour was detected in the position of the right kidney. In this case also, after nephrectomy, the other kidney quickly enlarged and became irregular in shape. The man died seven months after operation with the symptoms of pyelo-nephritis. The occurrence of cystic disease of the kidneys in two members of the same family is interesting. The writers quote Carl Beck's case. His patient was one of three sisters, each of whom suffered and died from this disease. Such examples point to a constitutional tendency to the disease affecting certain families. The average age of patients when first recognised to have the disease is about forty-five years. Usually its development is very slow, extending over several years, but the symptoms may be so slight that the kidney regions are not medically examined until enormous tumours have formed. The writers attribute the migrain in the first patient to the mobility of the kidney and the affection was cured, as in ordinary movable kidney, by nephropexy or nephrectomy. The attacks of severe lumbar pain have been variously explained as due to mobility of the kidney, sudden tension of the cyst contents, hæmorrhage into the cysts, or the passage of blood, mucus, crystals, or calculi down the ureter. Collis and Hewetson are convinced that sudden lumbar pain is occasionally due to rupture of cysts. This is borne out by the cicatrices seen on the surface of the kidneys in some specimens. Another case is mentioned in which when the sudden pain came on a fluid swelling formed in the lumbar region which ran forwards into the muscles of the abdominal wall. The swelling subsequently disappeared. The rapid increase of the opposite kidney after the diseased one has been removed is compensatory and shows that the diseased organ which was removed had not been functionally useless. Microscopical examination of the cystic organs removed shows that large areas of healthy and of partially healthy kidney tissue exist between the cysts. The authors consider nephrectomy to be contra-indicated in this disease. Even if the other kidney is healthy or in an early stage of the disease, it is more than likely that the increased demand made upon it must favour the development of cystic disease. The best results seem to be given by freely incising the diseased kidney. The larger cysts are systematically opened and packed with gauze. Thus the size of the organ and the pressure within its capsule are diminished and the remaining secreting tissue preserved.

[1] Brit. Med. Jour., June 17, 1905. [2] Lancet, May 20, 1905.

(*To be continued.*)

